# Layered Double Hydroxides Derived from MIL-88A(Fe) as an Efficient Adsorbent for Enhanced Removal of Lead (II) from Water

**DOI:** 10.3390/ijms232314556

**Published:** 2022-11-22

**Authors:** Jiang-Bo Huo, Guoce Yu

**Affiliations:** Laboratory of Environmental Technology, Institute of Nuclear and New Energy Technology, Tsinghua University, Beijing 100084, China

**Keywords:** MIL-88A(Fe), layered double hydroxides, core-shell structure, adsorption, Pb^2+^

## Abstract

The efficient removal of lead (II) from aqueous solution remains a big problem and the development of novel nanomaterials as adsorbents by various technologies to solve this problem is promising. This study contributed a novel nanostructure of MIL-88A-layered double hydroxides (LDHs) as the adsorbent for Pb^2+^, which was synthesized by a two-step solvothermal method with MIL-88A(Fe) as the precursor. The as-prepared material featured a chestnut-like core-shell structure, and exhibited excellent removal performance towards Pb^2+^ from water in comparison to MIL-88A(Fe) and LDHs (directly synthesized). The adsorption of Pb^2+^ by the MIL-88A-LDHs conformed to the pseudo-second-order kinetic model and the Langmuir and Freundlich isotherm models. The maximal adsorption capacity was 526.32, 625.00, and 909.09 mg g^−1^ at 278, 298, and 318 K, respectively. The thermodynamic parameters suggested that the adsorption was an endothermic, entropy-increasing, and spontaneous reaction. X-ray photoelectron spectroscopy (XPS) analysis indicated that the surface complexation was mostly responsible for Pb^2+^ elimination. The MIL-88A-LDHs can be readily regenerated and showed good cyclic performance towards Pb^2+^. Thus, the as-prepared MIL-88A-LDHs may hold promise for the elimination of aqueous heavy metals.

## 1. Introduction

Water pollution caused by heavy metals is of increasing concerned all over the world because of their easy accumulation and non-degradable properties in environmental media [1]. Once heavy metals are released into the environment and assimilated by life beings, their toxicity and carcinogenicity would be durable. Thus, efficient control of heavy metals pollution and reduction of their risks to human health is of importance for sustainable development of society and harmonious ecosystem. In general, metals with a density over 5 g/cm^3^ can be regarded as heavy metals [2]. There are a variety of heavy metals in the environment, such as Co, Ni, Cu, Zn, Cd, Hg, Cr, and Pb. Of them, Pb has been receiving much attention due to its high toxicity towards organisms and easy mobility in the media. Research has suggested excessive Pb^2+^ in blood would cause severe damage to the nervous system and sensory disturbance [3]. It is therefore imperative to enhance Pb^2+^ cleanup from water.

Various methods and technologies have been reported for the removal of Pb^2+^, mainly including chemical precipitation, membrane filtration, ion-exchange, adsorption and electrochemical technology. Among them, adsorption has been deemed as a popular and efficient method for removal of heavy metals thanks to the low cost, high efficiency, abundant sources of adsorbents, and easy manipulation. Until now, many materials have been designed and fabricated as adsorbents towards Pb^2+^. These adsorbents can be categorized into the following types: (i) natural minerals and clay-based adsorbents [4], (ii) carbon-based adsorbents, such as activated carbon, carbon fiber/tube, and graphene etc. [5,6], (iii) metal-based adsorbents, including metal oxides, metal sulfides, and layered double hydroxides etc. [7,8,9], (iv) metal-organic frameworks-based adsorbents [10], and (v) the hybrids (usually consisting of two or more components above) [11]. However, the adsorbents usually suffer from some limitations, e.g., the low adsorption capacity, the complex synthesis process, and the unsatisfactory cyclic performance, thus restricting their application. Considering the close relationship between structure and performance of adsorbents, the design and fabrication of novel adsorbents could pave the way towards boosting Pb^2+^ elimination [12].

Recently, metal-organic frameworks (MOFs) as templates or precursors for fabrication of porous materials such as metal oxides, metal carbides, and porous carbons have gained particular attention as an important research field [13,14]. MOFs-derived materials featured the periodic architecture with uniform pores and exhibited many unique properties. For instance, a composite with a structure of double-shelled nano-boxes was prepared by MOFs and it greatly enhanced oxygen evolution [15]. Magnetic carbon/iron composites were developed as an adsorbent for the efficient removal of tetracycline, which showed a high adsorption capacity (511.06 mg g^−1^) [16]. Moreover, MOFs-etched carbon/polymer membranes were reported for the high-performance extraction of organic pollutants [17].

However, studies concerning MOFs-derived layered double hydroxides (LDHs) have been rarely seen. LDHs, known as a two-dimension layer material, feature a cation layer structure with anions as the anticounter ions filling in the interlayer. Due to the large surface specific area, lamellar structure, and simple fabrication, LDHs and their composites have played important roles in removal of heavy metals. MOFs are usually built from (transition) metals and organic ligands, and LDHs are comprised of transition metals and inorganic anions. Can MOFs be in situ transformed into LDHs under suitable conditions? If so, the derivatives might possess some unique properties. Interestingly, some recent studies provided a support for the synthesis of such materials. For example, unique LDH nanocages with double shells from MIL-88A(Fe) precursor was designed for efficient oxygen evolution reaction [18]. A magnetic LDH composite from MIL-100(Fe) was synthesized for protein separation [19]. These reports confirmed the transformation of MOFs into LDHs and the unique structure of the resultant LDHs. So far, the study regarding the fabrication of LDHs derived from MOFs to remove heavy metals has not been reported yet. Thus, the development of novel LDHs with unique structure (e.g., a core-shell structure advantageous for Pb^2+^ adsorption) would be interesting and of significance for the cleanup of heavy metals.

In this study, we reported a chestnut-like (Ni, Fe) LDHs nanomaterial derived from MIL-88A(Fe), which was synthesized by a two-step solvothermal reaction. Its unique structure and properties were characterized by various analytical technologies. Applied as an adsorbent, its adsorption kinetics and isothermal behavior towards Pb^2+^ from water were investigated, and effects of solution initial pH and reaction temperature on the removal performance of Pb^2+^ were evaluated in batch adsorption. Additionally, the cyclic performance for Pb^2+^ cleanup and the adsorption mechanism were examined.

## 2. Results and Discussion

### 2.1. MIL-88A-LDHs Microstructure

**Figure 1:**
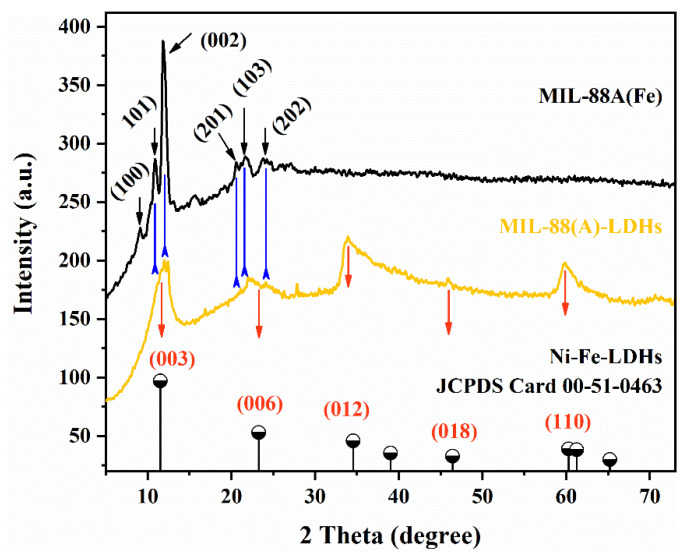
PXRD patterns of LDHs, MIL-88A(Fe), and MIL-88A-LDHs.

MIL-88A(Fe) precursors were fabricated by mixing fumaric acid and ferric chloride hexahydrate in DMF, and reacting at 100 °C for 8 h. As shown in Figure 1, after MIL-88A(Fe) reacted with urea and Ni^2+^, the characteristic peaks of MIL-88A(Fe) could be still recognized in MIL-88(A)-LDHs pattern, such as the planes of (002), (201), (103) and (202). However, the planes of (100) and (101) almost disappeared, which might be related to the reaction. Interestingly, several new peaks were also observed in the MIL-88(A)-LDHs pattern. By analyzing, the peaks around 11.5°, 23.2°, 34.5°, 46.4°, and 60.3° were ascribed to the planes of (003), (006), (012), (018), and (110), respectively. Those peaks can be well matched with those of Ni-Fe-LDHs (JCPDS Card 00-51-0462). This fact indicated that the LDHs phase was formed along the external surface of the MIL-88(A) phase by this protocol, leading to a core-shell nanostructure.

**Figure 2:**
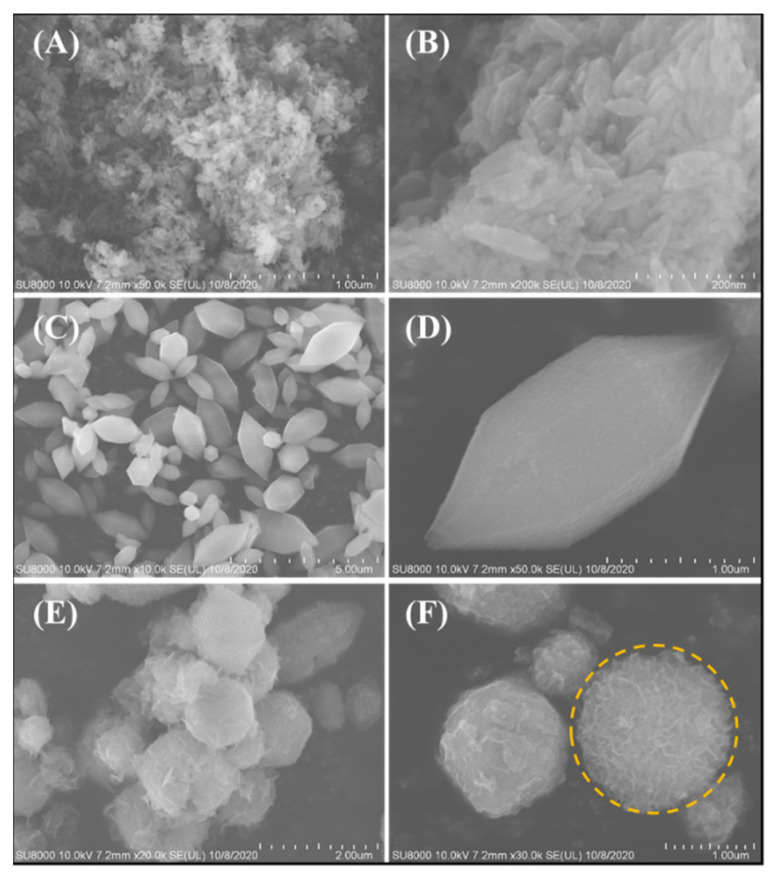
SEM images of (**A**,**B**) LDHs, (**C**,**D**) MIL-88A(Fe), and (**E**,**F**) MIL-88A-LDHs.

The SEM images of LDHs, MIL-88A(Fe), and MIL-88(A)-LDHs, are shown in Figure 2. It is easily found that LDHs consisted of many nanoplates, and these nanoplates suffered from a heavy agglomeration effect (Figure 2A,B). Distinct from LDHs, the pristine MIL-88A(Fe) had a hexagonal structure and topology with a length of ~2.2 µm and a width of ~1.2 µm (Figure 2C,D). In terms of morphology, MIL-88(A)-LDHs showed a unique hierarchical structure with a core of MIL-88A(Fe) and a shell of LDHs (Figure 2E,F). The yellow circle area may represent a top-view image of MIL-88(A)-LDHs subunit. Noticeably, the LDHs as shells were made up by much thinner nanosheets (~30 nm) than LDHs sheets (direct synthesis). This morphology may result from the role of MIL-88(A) template. During the synthesis process, the LDHs shells were gradually formed around the external surface of templates accompanied by the dissolution of MIL-88A(Fe). Influenced by steric hindrance, a hybrid with a unique core-shell structure was thus formed.

**Figure 3:**
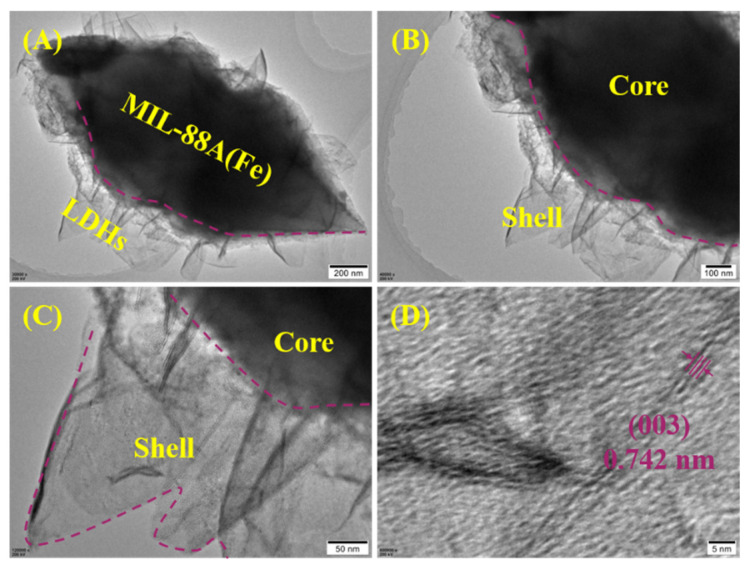
TEM images at different magnifications (**A**–**C**) and high-resolution TEM image (**D**) of MIL-88A-LDHs.

As shown in Figure 3, the TEM images showed a clear core-shell structure, in which the residual MIL-88A was a core (corresponding to the dark region) and the newly formed LDHs was a shell (corresponding to the light region) (Figure 3A–C). In the high-resolution TEM (Figure 3D), we can identify that interplanar spacing was approximately 0.742 nm, which roughly corresponded to the lattice plane of (003) of LDHs. This phenomenon was also identified by the PXRD analysis, and verified the formation of the LDHs phase.

### 2.2. Adsorption Performance

#### 2.2.1. Adsorption Kinetics

**Figure 4:**
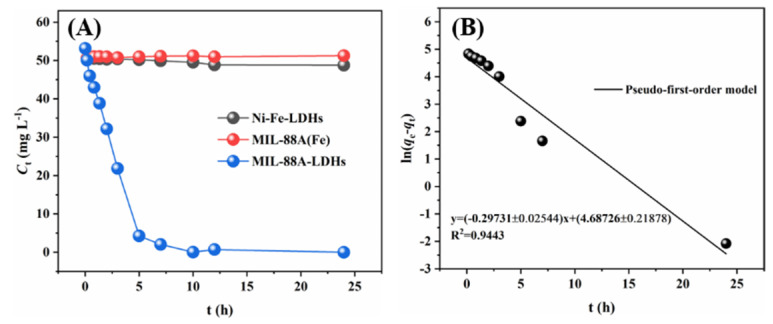

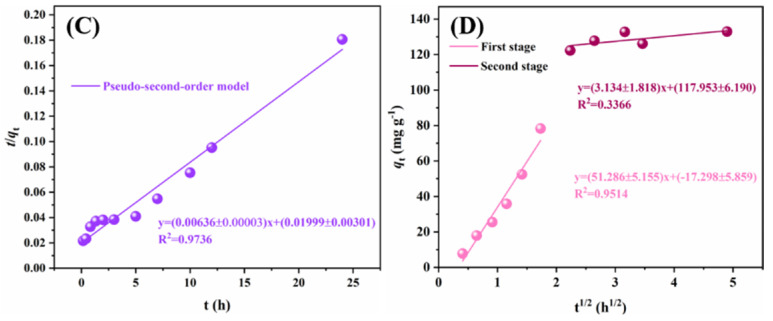
Adsorption of Pb^2+^ by LDHs, MIL-88A(Fe), and MIL-88A-LDHs: (**A**) effect of contact time, (**B**) PFO, (**C**) PSO and (**D**) intra-particle diffusion model. Experimental conditions: [Pb^2+^] = 53.15 mg L^−1^, initial pH = 3.0, adsorbent dose = 0.01 g, solution volume = 25 mL.

As shown in Figure 4A, MIL-88A-LDHs exhibited the best adsorption performance in comparison to Ni-Fe-LDHs and MIL-88A(Fe). Pb(II) concentration declined from 53.15 to 0.02 mg L^−1^, while for Ni-Fe-LDHs and MIL-88A(Fe), the concentration only decreased to 48.77 and 51.25 mg L^−1^, respectively. The remarkable capability of Pb(II) removal may have mainly originated from the unique core-shell structure of MIL-88A-LDHs. The shell enabled abundant active sites to be exposed to the metal ions.

For insight into adsorption behavior, three kinetic models were used to fit the experimental data. The models included pseudo first-order model (PFO) (Equation (1)), pseudo second-order model (PSO) (Equation (2)), and intraparticle diffusion model (Equation (3)), and the linear equations are listed as follows [20,21]:

ln(*q*_e_ − *q*_t_) = ln*q*_e_ − *k*_1_*t* (1)

*t*/*q*_t_ = 1/(*k*_2_*q*_e_^2^) + *t*/*q*_e_ (2)

*q*_t_ = *k*_i_·*t*^0.5^ + C (3)

where *q*_t_ and *q*_e_ (mg g^−1^) in Equations (1)–(3) represent the adsorption amount of Pb(II) at time of t and equilibrium, respectively. *k*_1_ (h^−1^), *k*_2_ [g (mg h)^−1^] and *k*_i_ [mg (g h^−0.5^)^−1^] are the rate constants of the pseudo first-order, the pseudo-second-order and the intraparticle diffusion models in Equations (1)–(3). The C (mg g^−1^) in Equation (3) reflects the effect of boundary layer on the adsorption.

Figure 4B,C show the fitting curves of two types of kinetic models and the corresponding parameter values are listed in Table 1. It can be easily found that both PFO and PSO fitted well with a high R-square of 0.9443 and 0.9736, respectively. PSO showed a better fitting result, indicating that the adsorption of Pb(II) by MIL-88A-LDHs might be dominated by chemisorption. It usually occurs via the ions exchange or the chemical reactions sharing electrons between adsorbents and adsorbates.

The intraparticle diffusion model was used to fit the adsorption process to identify the rate-limiting step. It was divided into two portions (Figure 4D). The first stage revealed the film diffusion through the boundary layer in the solid–liquid interface, while the second depicted the particle diffusion across the internal hydrated interlayer of adsorbents. The diffusion rate constants were 51.29 and 3.13 mg (g h^−0.5^)^−1^, respectively. These results suggested that the first stage was mostly controlled by the film diffusion process (a very small intercept) and the second almost depended on the intraparticle diffusion (a big intercept).

#### 2.2.2. Adsorption Isotherms

**Figure 5:**
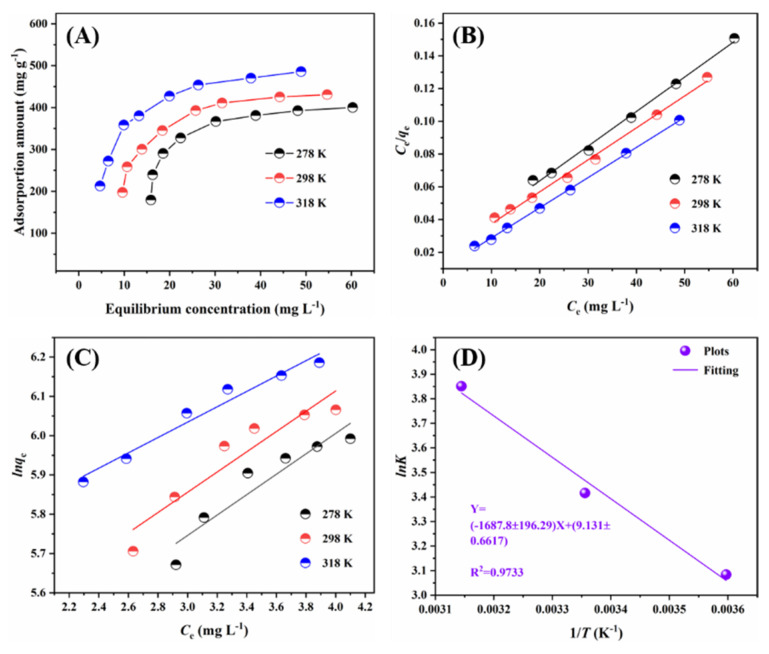
(**A**) adsorption isotherms at 278 K, 298 K and 318 K, (**B**) Langmuir isotherm model, (**C**) Freundlich isotherm model, and (**D**) Van Der Hoff equation. Experimental conditions: [Pb^2+^] = 85–280 mg L^−1^, initial pH = 3.0, adsorbent dose = 0.01 g, solution volume = 25 mL; t = 12 h.

The adsorption isotherms were investigated by changing the initial concentrations of Pb(II) at three temperatures (278 K, 298 K and 318 K). The curves are shown in Figure 5A. Obviously, the adsorption amount of Pb(II) increased with the equilibrium concentration of Pb(II) at each temperature level. Moreover, the adsorption amount showed an increasing trend as the temperature rose from 278 to 318 K. This implied that the adsorption was an endothermic reaction.

Moreover, we used the well-known isotherm models of the Langmuir (Equation (4)) and Freundlich (Equation (5)) equations to fit the adsorption data, and the linear form equations are given as follows [22]:

*C*_e_/*q*_e_ = 1/(*q*_m_*K*_L_) + *C*_e_/*q*_m_ (4)

ln*q*_e_ = 1/*n*·ln*C*_e_ + ln*K*_F_ (5)

where *C*_e_ (mg L^−1^) and q_e_ (mg g^−1^) are the equilibrium concentration of Pb(II) and the corresponding equilibrium adsorption amount, respectively. *K*_L_ (L mg^−1^) and *K*_F_ ((mg g^−1^)(L mg^−1^)1/*n*) are the constants of the Langmuir model and the Freundlich model, respectively.

The fitting curves are presented in Figure 5B,C and the calculated parameters are listed in Table 2. It was clear that the Langmuir model was better for fitting the adsorption of Pb(II) than the Freundlich model. This suggested a monolayer adsorption may occur between Pb(II) and MIL-88A-LDHs, which was driven by the chemical interactions [23].

A comparison with other sorbents is listed in Table 3. Obviously, the as-prepared MIL-88A-LDHs showed excellent adsorption capacity towards Pb(II) (512.8 mg g^−1^), which outperformed most of the newly synthesized adsorbents (below 200 mg g^−1^) [24,25,26,27,28].

#### 2.2.3. Adsorption Thermodynamic

To further investigate the thermodynamic features, the thermodynamic functions, such as enthalpy change (ΔH_0_, kJ/mol), entropy change (ΔS_0_, J/(mol·K)), and Gibb’s free energy change (ΔG_0_, kJ/mol), were calculated from Equations (6) and (7):

∆G_0_ = −*RT*lnK_0_ (6)

lnK_0_ = ∆S_0_/*R* − ∆H_0_/*RT* (7)

where *R* is the ideal gas constant, 8.314 J/mol/K; *T* is the Kelvin temperature, K; K_0_ represents the thermodynamic equilibrium constant and lnK_0_ can be obtained by the extrapolation of ln(*q*_e_/*C*_e_) vs. *C*_e_.

The Van Der Hoff curve is shown in Figure 5D and the thermodynamic parameters are provided in Table 4. The negative values of ΔG_0_ indicated the adsorption of Pb(II) by MIL-88A-LDHs was spontaneous. In addition, the absolute value of ΔG_0_ increased with the elevated temperature, revealing that high temperature can boost the adsorption reaction. The values of ΔH_0_ and ΔS_0_ were calculated to be 14.03 kJ/mol and 75.91 J/mol/K respectively, based on the intercept and slope of Van Der Hoff equation (Figure 5D). The positive value of ΔH_0_ suggested that Pb(II) adsorption by MIL-88A-LDHs was an endothermic process, whereas the positive value of ΔS_0_ revealed an increase in randomness at interface. Thus, the adsorption was an endothermic, entropy-increasing, and spontaneous process [3,29].

#### 2.2.4. Effects of Adsorbent Dosage and pH

**Figure 6:**
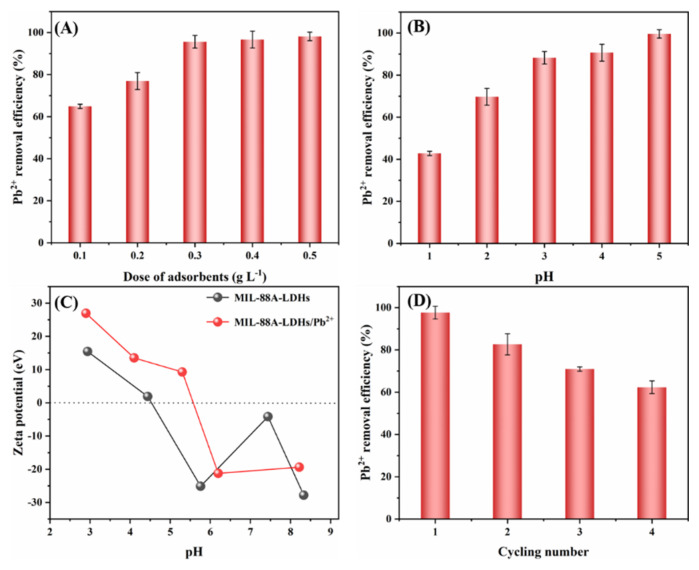
Effects of (**A**) adsorbent dosage and (**B**) solution initial pH. Experimental conditions: [Pb^2+^] = 88 mg L^−1^, initial pH = 3.0, adsorbent dosage = 0.01 g, solution volume = 25 mL; t = 12 h. (**C**) zeta potential of MIL-88A-LDHs and MIL-88A-LDHs/Pb^2+^. (**D**) Cyclic performance of MIL-88A-LDHs.

The effects of adsorbent dosages on Pb(II) removal were investigated and the results are shown in Figure 6A. The removal percentages increased with increasing the dosage. When the dosages varied from 0.1 to 0.5 g/L, the Pb(II) removal percentages increased from 64.92 to 98.16%. This could be due to the presence of more available active sites at a higher dosage in the solution. However, the unoccupied adsorption sites would no longer make contributions to the adsorption when the dosage exceeded the optimal value [30]. The dosage of 0.4 g/L was fixed in this study, since the removal percentage can reach 96.66% at the dosage.

As shown in Figure 6B, the Pb(II) adsorption depended heavily on the solution pH, and the removal percentage obviously increased from 42.77 to 99.59% when pH increased from 1.0 to 5.0. Additionally, the point of zero charge (pHzpc) of MIL-88A-LDHs was about 4.6 (Figure 6C). A relatively high removal efficiency (~90.68%) was achieved at pH < pHzpc, and this phenomenon suggested the electrostatic interaction was not the only driving force. Given the constituents of MIL-88A-LDHs, it can be inferred that the precipitation between lead ions and anions (OH^−^ or CO_3_^2−^) may make an important contribution to the adsorption.

#### 2.2.5. Adsorption Mechanism

**Figure 7:**
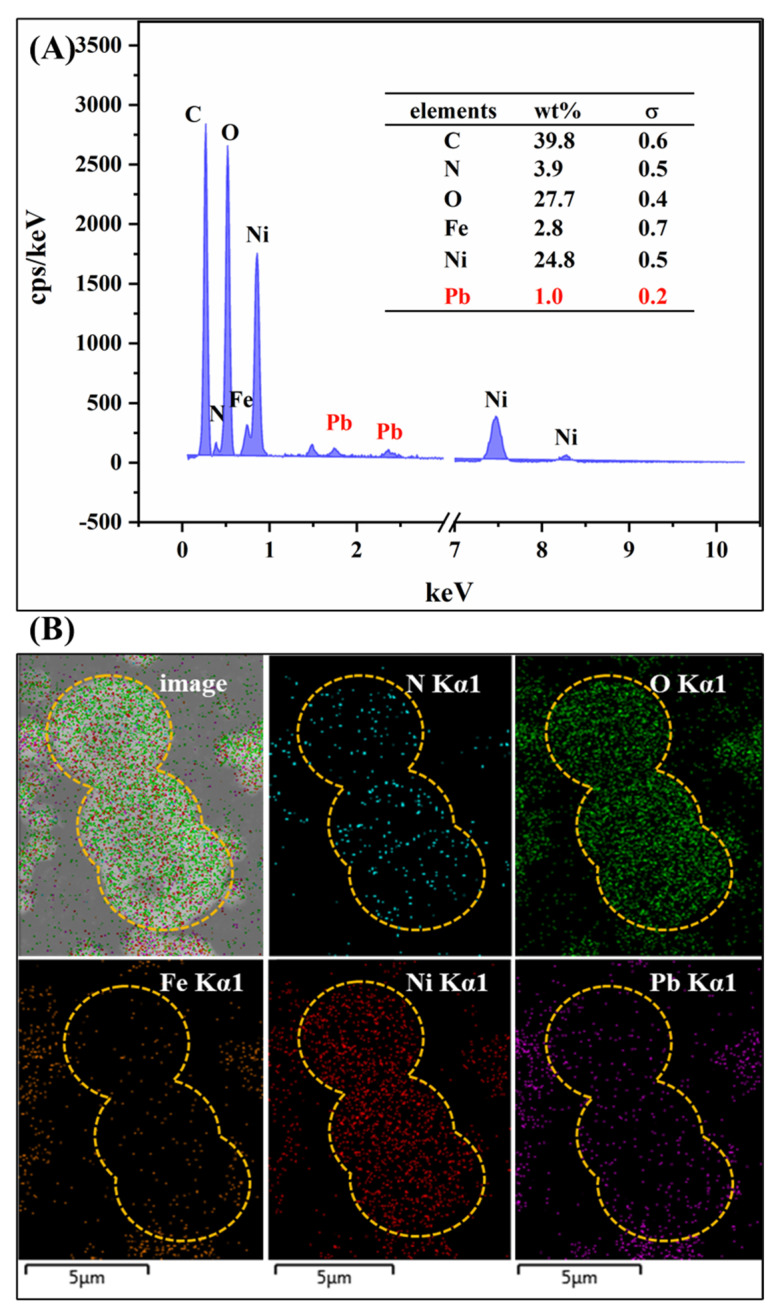
(**A**) SEM-EDX and (**B**) element mapping of MIL-88A-LDHs/Pb^2+^.

The SEM-EDX spectrum and elemental mapping are shown in Figure 7. After adsorption, the Pb signals have been detected in the used adsorbent surface and the contents were approximately 1.0 wt%. In addition, the elemental distributions were almost identical. These findings evidenced that Pb(II) had successfully attached to the adsorbent surface.

**Figure 8:**
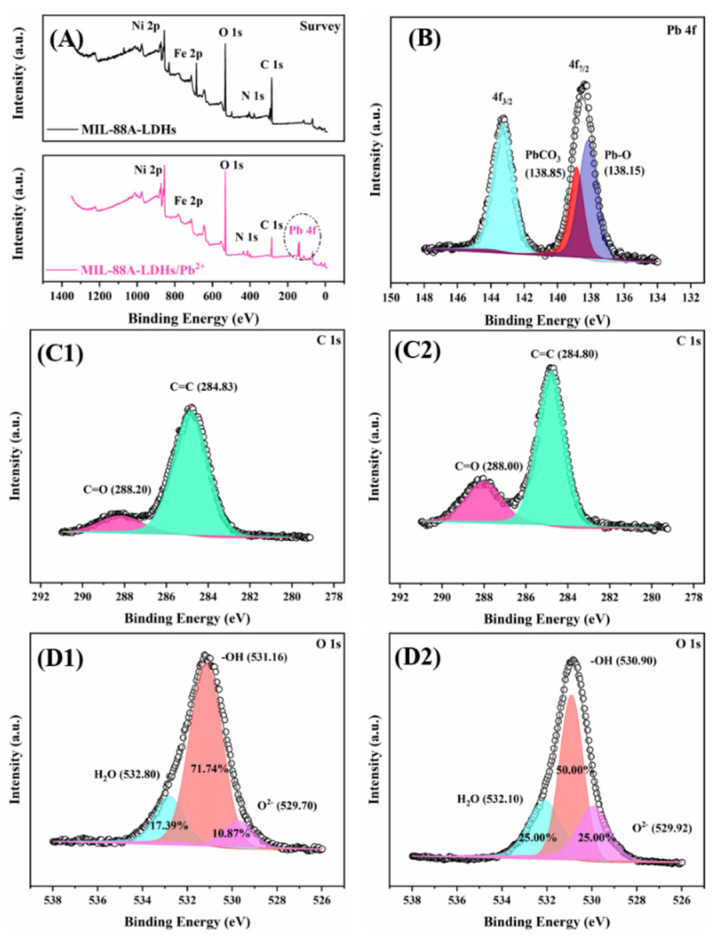
XPS spectra of MIL-88A-LDHs and MIL-88A-LDHs/Pb^2+^: (**A**) survey, (**B**) Pb 4f, (**C1**,**C2**) C 1s, (**D1**,**D2**) O1s.

XPS can provide the information of chemical states of surface elements for the purpose of unveiling the adsorption mechanism. As depicted in Figure 8A, the main elements included Ni, Fe, O, N, and C in the survey spectra of two samples. Particularly, the Pb 4f characteristic peak was found in the MIL-88A-LDHs/Pb^2+^. The Pb chemical states can be divided into two main species PbCO_3_ and Pb-O in the high resolution Pb 4f spectrum (Figure 8B), and their locations were approximately at 138.85 eV and 138.15 eV, respectively. These results definitely confirmed the successful adsorption of Pb(II) by MIL-88A-LDHs.

The high-resolution C 1s spectra of MIL-88A-LDHs and MIL-88A-LDHs/Pb^2+^ were deconvoluted into two peaks, i.e., C=O and C=C, as shown in Figure 8C1,C2. It can be found that the relative contents (peak area) changed and peak locations slightly shifted to low binding energy orientation (from 288.20 and 284.83 eV to 288.00 and 284.80 eV, respectively). These phenomena resulted from the formation of PbCO_3_. CO_3_^2−^ went through the internal pore to reach the interface and encountered Pb(II) to form precipitate. Thus, the contents of surface Pb(II) obviously increased and the peak movement may be caused by the strong interaction between Pb(II) and CO_3_^2-^. This observation further verified that Pb(II) was removed by the formation of precipitate.

Moreover, the high-resolution O1s spectra of two samples were divided into three peaks. As shown in Figure 8D1,D2, the relative contents of −OH species heavily decreased to 50.00% from 71.74% (before adsorption), while those of H_2_O and O^2−^ species showed an increasing trend (up to 25.00% from 17.39% and 10.87%, respectively). This result suggested that there were abundant OH− (LDHs structural constituents) or surface hydroxyls consumed during the Pb(II) adsorption process, and confirmed that the surface hydroxyls also served as adsorption sites for Pb(II) cleanup, besides the precipitation of CO_3_^2−^ [31,32,33].

**Figure 9:**
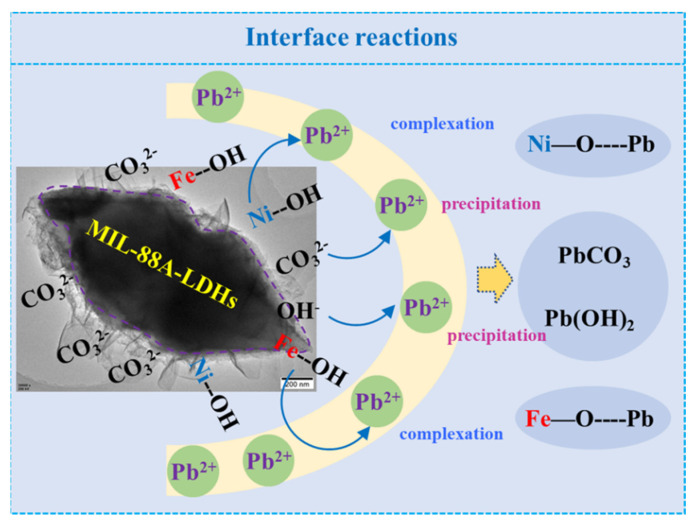
Schematic illustration of the mechanism of Pb^2+^ adsorption by MIL-88A-LDHs.

By the analysis above, we can draw a scheme of adsorption mechanisms (Figure 9). Both CO_3_^2−^ and surface hydroxyls made contributions to Pb(II) elimination. After adsorption, Pb(II) existed as PbCO_3_, Pb(OH)_2_, and Pb-complex on the adsorbent surface.

### 2.3. Cycling Performance of MIL-88A-LDHs

The cycling performance of MIL-88A-LDHs has been investigated. As shown in Figure 6D, the Pb(II) removal efficiency dropped from 97.64% at the first cycle to 62.35% at the fourth cycle. The as-prepared adsorbent only maintained moderate adsorption ability since some active sites occupied by Pb(II) could not be regenerated. Particularly, the PbCO_3_ precipitation would consume CO_3_^2−^. The exploration of a regenerable adsorbent with excellent adsorption capacity is still ongoing.

## 3. Materials and Methods

### 3.1. Materials and Reagents

All chemicals were of analytical grade. Dimethylformamide (DMF), ethanol, fumaric acid (FA), ferric chloride hexahydrate (FeCl_3_·6H_2_O), nickel nitrate hexahydrate (Ni(NO_3_)_2_·6H_2_O), urea, citric acid, and lead nitrate (Pb(NO_3_)_2_) were purchased from Sinopharm Chemical Reagent Co., Ltd (Shanghai, China).

### 3.2. Synthesis of MIL-88A(Fe), Layered Double Hydroxides (LDHs), and LDHs Derived from MIL-88A(Fe)

#### 3.2.1. Synthesis of MIL-88A(Fe)

Typically, 0.928 g of FA and 1.296 g of FeCl_3_·6H_2_O were dissolved in 80 mL DMF. The mixture in a Teflon-lined stainless-steel autoclave (100 mL) was transferred to an electric oven to react at 100 °C for 8 h. After reaction, the precipitate was collected by centrifugation and washed by absolute ethanol and ultrapure water repeatedly. Finally, the product was dried overnight at 80 °C for further use.

#### 3.2.2. Synthesis of Layered Double Hydroxides (LDHs)

Typically, 1.5 g of ferric chloride hexahydrate, 0.52 g of nickel nitrate hexahydrate, and 1.0 g of urea were dissolved in 60 mL deionized water and reacted at 90 °C for 12 h. After reaction, the precipitate was collected by centrifugation and washed by ultrapure water repeatedly. Finally, the product was dried overnight at 80 °C for further use.

#### 3.2.3. Synthesis of LDHs Derived from MIL-88A(Fe)

**Figure 10:**
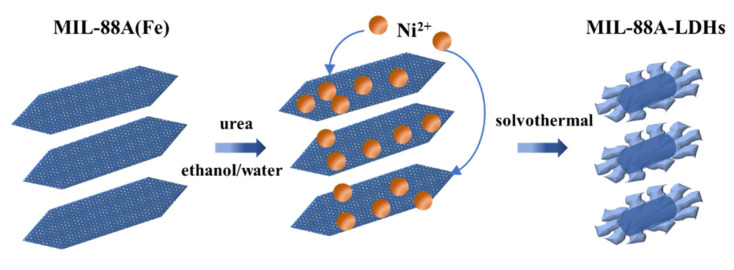
Schematic illustration of the synthesis process of MIL-88A-LDHs.

The scheme of synthesis of LDHs derived from MIL-88A(Fe) is shown in Figure 10. Firstly, 80 mg of MIL-88A(Fe) was dissolved in 48 mL ethanol, labelled as solution A. Then 1.2 g of nickel nitrate hexahydrate and 0.8 g of urea were dissolved in 32 mL deionized water, labelled as solution B. Solution A and B were mixed to form a homogeneous mixture. The mixture was poured into a Teflon-lined stainless-steel autoclave (100 mL) and transferred to an electric oven to react at 95 °C for 6 h. After reaction, the precipitate was collected by centrifugation and washed by absolute ethanol and ultrapure water repeatedly. Finally, the product was dried overnight at 80 °C for further use. The product was denoted as MIL-88A-LDHs.

### 3.3. Characterization of Materials

Powder X-ray diffraction (XRD) patterns of the samples were recorded with a X’Pert Pro diffractometer (PANalytical, Eindhoven, Holland) using Cu Kα radiation (40 kV, 40 mA, λ = 0.15406 nm). The surface morphology of the materials was observed using a scanning electron microscope (SEM, Hitachi SU-8010, Tokyo, Japan) equipped with an energy dispersive X-ray spectrometer (EDS). The structure and the elemental distribution were determined using a transmission electron microscope (TEM, JEOL, JEM-2100F, Tokyo, Japan) operated at 200 kV. X-ray photoelectron spectroscopy (XPS) was performed in a PHI 5000C ESCA system (PHI, Louisiana, USA). The lead contents in the samples were measured quantitatively by atomic absorption spectroscopy (AAS) by using a HITACHI ZA3000 instrument (Tokyo, Japan).

### 3.4. Adsorption Experiments

The adsorption behavior of MIL-LDHs was evaluated by batch adsorption experiments to determine the kinetics, equilibrium and thermodynamic experimental results. All the experiments were conducted in 50 mL plastic vials with each containing 25 mL solution. The dosages of MIL-88(A), LDHs, and MIL-LDHs used in Pb(II) adsorption experiments were set as 0.4 g L^−1^. All solution samples were shaken in a ZHWY-2102C incubator shaker (ZhiCheng, Shanghai, China) with the desired temperature of 25 °C and fixed rotating speed of 250 rpm in the sorption process.

The adsorption kinetic experiments of Pb(II) on MIL-88(A), LDHs, and MIL-LDHs were all performed at an initial concentration of 53.15 mg L^−1^ and shaken for 12 h in the incubator shaker. At predetermined time intervals, replicate vials were sacrificially sampled to measure the residual concentration of Pb(II) in the liquid phase. The pseudo-first-order model and the pseudo second-order model were employed to fit kinetic data for further analysis.

The adsorption isotherm experiments of Pb(II) on MIL-LDHs at three temperatures (5, 25 and 45 °C) were all established with concentrations ranging from 85 to 280 mg L^−1^ after a 24 h oscillation. The Langmuir and Freundlich models were used to fit isotherm data for further analysis.

By varying from 0.1 to 0.5 g L^−1^, effects of adsorbent dosages on Pb(II) removal efficiency were performed. The initial Pb(II) concentration was 88 mg L^−1^ and the solution pH was adjusted to 3.0. The pH effects on Pb(II) removal efficiency were investigated at a pH range of 1.0–5.0. The adsorbent dosage was 0.4 g L^−1^.

### 3.5. Cycling Experiments

The adsorption experiments were performed under the condition of the initial Pb(II) concentration of 20 mg L^−1^ and the dosage of 0.4 g L^−1^. After equilibrium, the used MIL-LDHs were recovered by filtration, and were redispersed in the citric acid solution (0.02 mol/L) for desorption. The MIL-LDHs were rinsed by ethanol and deionized water successively. After drying treatment (105 °C, 8 h), the regenerated MIL-LDHs were used for next cycle.

All collected filtrates were measured by polarized Zeeman Atomic Absorption Spectrophotometer ZA3000 Series (AAS, Hitachi, Tokyo, Japan). In this study, all adsorption experiments were conducted in duplicate and the average data were reported. The removal percentage (R) and the adsorption amount were calculated from the following Equations (8) and (9):

*R* = ((*C*_0_ − *C*_e_))/*C*_0_ × 100% (8)

*q*_t_ = ((*C*_0_ − *C*_t_) × V)/m (9)

where *C*_0_, *C*_t_, and *C*_e_ (mg L^−1^) are the Pb(II) initial concentration, the instant concentration at time t and the equilibrium concentration in the solution, respectively. V (mL) represents the volume of the Pb(II) solution, and m (mg) the mass of the adsorbents.

## 4. Conclusions

This study contributed a novel adsorbent for Pb^2+^ of MIL-88A-layered double hydroxides (LDHs), which was synthesized by a two-step solvothermal method with MIL-88A(Fe) as the precursor. The as-prepared material featured a chestnut-like unique structure and exhibited excellent removal performance towards Pb^2+^ from water in comparison to MIL-88A(Fe) and LDHs (directly synthesized). The adsorption of Pb^2+^ by the MIL-88A-LDHs was well fitted by the pseudo-second-order kinetic model and the Langmuir and Freundlich isotherm models. The maximal adsorption capacity was 526.32, 625.00, and 909.09 mg g^−1^ at 278, 298, and 318 K, respectively. The thermodynamic parameters suggested that the adsorption was an endothermic, entropy-increasing, and spontaneous reaction. XPS analysis indicated that the surface complex model was mostly responsible for Pb^2+^ elimination. The MIL-88A-LDHs can be readily regenerated and showed good cyclic performance towards Pb^2+^. Thus, the MIL-88A-LDHs may be taken as a promising adsorbent for removing Pb^2+^ and other heavy metals from water.

## Figures and Tables

**Table 1:** Adsorption kinetic parameters.

PFO		PSO		Intra-Particle Diffusion	
*R* ^2^	0.9443	*R* ^2^	0.9736	*R* ^2^	0.9512
*k*_1_	0.2971	*k*_2_	0.0020	*k*_i1_	51.29
*q*_e_	108.5	*q*_e_	157.2	*q*_e_	17.30
				*R* ^2^	0.3366
				*k*_i2_	3.13
				*q*_e_	117.95

Notes: *k*_1_ (h^−1^), *k*_2_ [g (mg h)^−1^] and *k*_i_ [mg (g h^−0.5^)^−1^]; *q*_e_ (mg g^−1^).

**Table 2:** Adsorption isotherm parameters.

	Langmuir Model			Freundlich Model		
	*K*_L_	*R*_2_	*q*_m_	*K*_F_	*R* ^2^	*n*
278 K	0.0983	0.9945	473.9	143.6	0.8665	3.853
298 K	0.1087	0.9946	512.8	161.2	0.8682	3.881
318 K	0.1767	0.9989	649.4	232.3	0.9591	5.117

Note: *K*_L_: (L mg^−1^), *K*_F_: ((mg g^−1^)(L mg^−1^)1/n), and *q*_m_ (mg g^−1^).

**Table 3:** Comparison of adsorption capacity of newly reported Pb(II) adsorbents.

No.	Adsorbent	pH	Maximum Capacity (mg g^−1^)	Ref
1	Co-Fe_2_O_3_/Ni-Fe_2_O_3_	5.0	136.0/97.5	[24]
2	Fe_3_O_4_-Cu-MOF	4.0	610.0	[25]
3	HT NPs	-	169.5	[26]
4	Epsilon-MnO_2_	-	239.7	[27]
5	Modified biochar	5.0	145.0	[28]
6	MIL-88A-LDHs	3.0	512.8	This study

**Table 4:** Adsorption thermodynamic parameters.

Temperature (K)	ΔG_0_ (kJ mol^−1^)	ΔH_0_ (kJ mol^−1^)	ΔS_0_ (J mol^−1^ K^−1^)
278	−7.128	14.03	75.91
298	−8.463		
318	−10.18		

## Data Availability

Not applicable.
